# Valorization of Apple Pomace: Production of Phloretin Using a Bacterial Cellulose‐Immobilized β‐Glycosidase

**DOI:** 10.1002/cssc.202500592

**Published:** 2025-04-30

**Authors:** Agostina Colacicco, Luca Nespoli, Emma Ribul Moro, Stefano Farris, Francesco Molinari, Diego Romano, Martina Letizia Contente

**Affiliations:** ^1^ Department of Food, Environmental and Nutritional Sciences (DeFENS) University of Milan via Celoria, 2 20133 Milan Italy

**Keywords:** apple processing byproducts, bacterial cellulose, biocatalysis, enzyme immobilization, phloretin

## Abstract

In the last decade, phloretin (PHL) has attracted increasing attention due to its remarkable biological properties, including antimicrobial, antidiabetic, cardioprotective, anti‐inflammatory, immunomodulatory, and antioxidant effects, becoming a leading ingredient in the cosmetic sector. In this work, an efficient, cost‐effective, and highly productive biocatalytic strategy for the preparation of natural PHL has been developed starting from its glycosylated form, phloridzin, one of the main flavonoid components of apple processing waste (apple pomace). The process involved the use of the extremophilic β‐glycosidase *AHe*GH1 immobilized on bacterial cellulose films in a two‐liquid phase reaction system (water/2,2,5,5‐tetramethyloxolane), allowing for the complete conversion of 5 g L^−1^ of substrate in 7 h of reaction (molar conversion >99%; isolated yield 95%). Since all the materials used in the biotransformation have been recovered and recycled (i.e., solvents, aqueous phase, and catalyst), this system can be considered a zero‐waste reaction. Interestingly, a further leap forward in the overall bioprocess sustainability was achieved by producing bacterial cellulose, the support for enzyme immobilization, by fermentation of apple pomace. This allows for a biocatalytic process where both the substrate and the immobilization carrier derive from the same feedstock.

## Graphical abstract

This study presents a whole valorization of apple processing waste: phloretin is efficiently produced from phloridzin (abundantly found in apple residues), using a β‐glycosidase immobilized on bacterial cellulose in a biphasic system. Notably, bacterial cellulose is derived from fermentation of apple pomace sugars, making both the enzyme carrier and substrate originate from the same feedstock in a sustainable process. ,

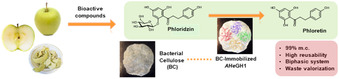

## 1 Introduction

**Scheme 1:**
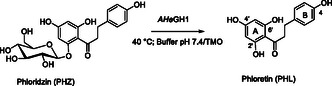
Preparation of natural PHL *via* the extremophilic β‐glycosidase *AHe*GH1.

In the last ten years, particular attention has been paid to phloretin (PHL), a naturally occurring flavonoid mainly found in the non‐edible parts of *Malus* genus apple trees (e.g., skin, stem, flesh, seed, and leaves) as glycosylated form (i.e., phloridzin, PHZ, **Scheme** **1**).^[^ ^1^ ^]^ PHL is a dihydrochalcone formed by two aromatic rings (A and B), bearing hydroxyl groups in 2’, 4’, 6’, and 4 positions and connected to a three‐carbon α,β‐unsaturated carbonyl moiety (Scheme 1). Its remarkable structure has been associated with a variety of biological properties, including antimicrobial,^[^ ^2^, ^3^ ^]^ antidiabetic,^[^ ^4^, ^5^, ^6^ ^]^ cardioprotective,^[^ ^7^, ^8^ ^]^ anti‐inflammatory,^[^ ^9^, ^10^ ^]^ immunomodulatory^[^ ^11^ ^]^ and antiaging effects.^[^ ^12^ ^]^

The importance of the free hydroxyl group in position 2’ has been highlighted to further enhance the antibacterial activity, especially against gram‐positive strains (e.g., *Staphylococcus aureus* and *Listeria monocytogenes*)^[^ ^2^ ^]^ as well as the antioxidant capacity.^[^ ^13^ ^]^ Furthermore, the reduced polarity of the aglycone with respect to the glycosylate for increased PHL processability in pharmaceutical and cosmetic preparations. A practical example has been reported by L’Oreal USA, where PHL formulated with vitamin C and ferulic acid has been used as a skin lightening agent and UV protector.^[^ ^14^ ^]^ In this context, PHL serves a dual purpose: as an inhibitor of tyrosinase, the key enzyme of skin pigmentary disorders, and as a stabilizer of the formulation, facilitating the skin penetration of the other components.^[^ ^12^, ^15^ ^]^ In fact, due to PHL ability to interact with bilayer membranes, increasing their fluidity, it can be employed as a skin penetration enhancer.

Currently, apple pomace, a mixture of residues and byproducts deriving from apple processing such as skin, stem, flesh, seed, and leaves (counting around 4 million metric tons produced every year), has attracted increasing attention.^[^ ^16^ ^]^ On one hand, apples, being the most important dietary source of polyphenols, are widely available worldwide, thus facilitating the recovery of their processing waste.^[^ ^17^, ^18^ ^]^ On the other hand, given that apple pomace poses diverse adverse effects on the environment, its valorization can mitigate the costs associated with its management and disposal. Moreover, the high content of valuable phenolic compounds in apple residues^[^ ^16^, ^19^ ^]^ makes this waste material a cost‐effective natural source of bioactive molecules with potential applications in the pharmaceutical, cosmetic, and food industries. Utilizing these residues not only reduces biowaste but also creates new economic opportunities by transforming underutilized materials into high‐value products.

The small amount of PHL present in the biomass with respect to its glycoside (0.1%–1%, 10%–14% of leaf dry weight, respectively),^[^ ^1^ ^]^ leads us to focus on the more available PHZ as a starting point for the preparation of PHL. Furthermore, PHZ extraction from apple pomace *via* green technologies has already been described in several reports.^[^ ^1^, ^19^, ^20^, ^21^, ^22^, ^23^, ^24^ ^]^

In the design of green processes, biocatalyzed approaches have been recognized as a smart tool to enhance the sustainability of chemical reactions.^[^ ^25^, ^26^, ^27^, ^28^ ^]^ In this context, extremophilic β‐glycosidases proved to be robust enzymes particularly suited to harsh conditions, naturally tolerating organic solvents, drastic temperatures, high sugar concentration, etc.^[^ ^29^, ^30^, ^31^, ^32^ ^]^ Herein, the β‐glycosidase *AHe*GH1 from the thermo‐acidophilic organism *Alicyclobacillus herbarius* (*AHe*)^[^ ^33^ ^]^ was employed as an efficient catalyst for the preparation of PHL from PHZ (Scheme 1). Conversely, chemical attempts to obtain pure flavonoids mainly rely on hydrolysis reactions starting from the corresponding glycosides or *ex novo* strategies for their total synthesis. Both processes are usually characterized by low yields, long procedures (e.g., protection/deprotection steps), and difficult purifications due to the presence of side products (e.g., intramolecular cyclization, polymerization).^[^ ^34^, ^35^, ^36^, ^37^ ^]^

Although biocatalysis has some advantages over conventional chemistry in terms of higher selectivity, milder operational conditions, involvement of less energy consuming and generally safer processes,^[^ ^25^, ^28^, ^38^, ^39^ ^]^ just a few examples employing commercial hydrolytic enzymatic preparations have been reported for the synthesis of PHL.^[^ ^40^, ^41^ ^]^ Unfortunately, the above mentioned enzymatic procedures are characterized by diluted solutions, low substrate loading, and poor yields, thus limiting their employment on a preparative scale.^[^ ^40^, ^41^ ^]^ On the other hand, the homemade production of proteins is typically considered a cost and time‐consuming technique, especially when the application of pure enzymes is limited by their low operational stability and reusability. Thus, enzyme immobilization technology is considered a key strategy to overcome these limitations.^[^ ^42^, ^43^, ^44^, ^45^, ^46^, ^47^ ^]^ Immobilization of enzymes not only enhances the catalyst stability, but also its recovery and reuse, thus facilitating the downstream processing while reducing the process related costs. Among the carriers for protein immobilization, cellulose exhibits several advantages, such as availability, biodegradability, biocompatibility, low cost, and chemically inert behavior.^[^ ^48^ ^]^ In this context, bacterial cellulose (BC) produced *via* microbial fermentation (i.e., using acetic acid bacteria ‐AAB‐) particularly fits the sustainability aim, as it can be easily produced from rich‐in‐sugar residues and byproducts (e.g., agri‐food waste),^[^ ^49^, ^50^ ^]^ especially coming from the fruit transformation industry.^[^ ^51^ ^]^ Recent studies have investigated the use of these biomasses as a medium for BC production. However, many approaches require pretreatment of agro‐waste to enhance nutrient availability, extended fermentation times (>10 days), or the addition of a nitrogen source to boost BC production.^[^ ^52^, ^53^ ^]^

Various BC forms have been successfully explored as supports for enzyme immobilization, particularly for lipases and a selected group of other hydrolases. These include nanocellulose^[^ ^54^, ^55^ ^]^ nanocrystals,^[^ ^56^ ^]^ sphere‐like structures,^[^ ^57^ ^]^ and magnetic‐composite carriers.^[^ ^58^ ^]^ Most of these immobilization systems rely on simple adsorption onto BC supports, leveraging its high surface area. However, this approach often leads to enzyme leakage, limiting the system reusability.^[^ ^59^ ^]^

Other BC‐based immobilization strategies involving a stable covalent bonding between the enzyme and the support are achieved through chemical modification of BC using coupling agents such as 1‐ethyl‐3‐(3‐dimethylaminopropyl)carbodiimide (EDC), 1,4‐butanediol diglycidyl ether, or glutaraldehyde.^[^ ^54^, ^57^ ^]^ Covalent immobilization enhances the reusability of the system by minimizing enzyme detachment while maintaining a good residual activity. In this context, Wu et al.^[^ ^60^ ^]^ investigated the use of BC pellets for amylase immobilization. However, their approach showed limited immobilization efficiency and revealed that pellet size significantly impacted enzyme activity retention.

In one of the most recent publications, Panagopoulos and coworkers reported the immobilization of a β‐galactosidase and a glucose isomerase onto BC membranes, indicating improved productivity of fructose and lactulose from lactose, compared to their free enzyme counterparts.^[^ ^61^ ^]^ However, the study provides limited details on aspects such as the immobilization mechanism, potential protein leakage over time, operational stability, and reusability of the immobilized catalyst—important factors to evaluate the system applicability and efficiency.

In this work, we developed an efficient, cost‐effective, and highly productive preparation of natural PHL from PHZ, employing the extremophilic *AHe*GH1 covalently immobilized onto BC pellicles. Due to the increased stability of our system, not only water media biotransformations have been performed, but also biphasic systems (organic solvent:water 50:50) were set up, allowing for a continuous and facile removal of the desired product and the collection of the sugar moieties in the water phase for their potential reuse. Moreover, the immobilized *AHe*GH1 on BC pellicles could be reemployed for 13 consecutive reaction cycles, retaining more than 40% of its initial activity. To further valorize the apple pomace, this residue was employed as fermentation feedstock for BC production without any pretreatment and nitrogen source addition. The developed process represents a good example of complete reutilization and valorization of residues from apple processing. Moreover, according to the regulations of the FDA (i.e., Food and Drug Administration) and the EMA (i.e., European Medicinal Agency), processing natural starting materials with biocatalysts allows for the commercialization of the final product as natural, thereby increasing its market value.

## 2 Results and Discussion

### 2.1 Free Enzyme Biotransformations

Based on our experience on the use of extremophilic β‐glycosidases,^[^ ^29^, ^30^, ^31^ ^]^ the batch‐biotransformation for the production of PHL from PHZ has been carried out following our previous reported optimized conditions (5 g L^−1^ substrate, 1 mg mL^−1^ enzyme, in 4‐(2‐hydroxyethyl)‐1‐piperazineethanesulfonic acid (HEPES) buffer pH 7.4, 10% *v/v* dimethyl sulfoxide (DMSO) at 40 °C). Complete conversion of PHZ into PHL has been obtained after 24 h of reaction, corresponding to a space time yield (STY) of 1 g L^−1^ h^−1^ (see Supporting Information). To favor the solubility of both the substrate and product, avoiding the addition of DMSO in the reaction environment, biphasic transformations employing a water‐immiscible green solvent 2,2,5,5‐tetramethyloxolane (TMO)^[^ ^62^ ^]^ have been set up (i.e., 50:50 water/TMO, 1 mg mL^−1^ catalyst concentration, 5 g L^−1^ substrate loading). TMO has demonstrated to be a valid greener alternative to toluene in lipase‐mediated polymerization reactions, as well as to be well tolerated by enzymes, especially extremophilic ones.^[^ ^31^ ^]^ After 24 h of *AHe*GH1‐mediated biotransformation, PHL has been obtained with >99% m.c. (see Supporting Information). Noteworthy, the use of TMO dramatically facilitated the downstream processing and the recovery of the desired product, which was isolated in 95% yield.

### 2.2 Phloretin Preparation *via* Bacterial Cellulose‐Immobilized *AHe*GH1

**Figure 1:**
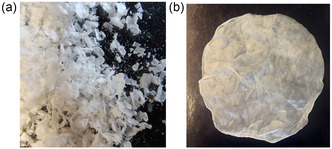
Different BC forms used in this works: a) BC powder and b) BC pellicles.

To enhance the cost‐efficiency of the reaction together with the catalyst stability under operational conditions (i.e., biphasic media, 40 °C, 5 g L^−1^ PHZ concentration), a tailored immobilization of *AHe*GH1 (imm‐*AHe*GH1) was developed. A covalent bond between the matrix and the enzyme has been selected to specifically obtain robust and durable catalysts to be (re)used for different reaction cycles without enzyme leaking. BC produced by *Komagataeibacter xylinus* DSM2325 and previously functionalized with APTES (3‐aminopropyl)triethoxysilane) and glutaraldehyde (see Supporting Information) has been chosen as support for its unique structure and properties such as chemical purity, nanoscale fibrous 3D network, high degree of polymerization, high crystallinity, and mechanical features.^[^ ^63^, ^64^ ^]^ Moreover, obtaining high‐purity BC involves a simpler process than obtaining plant‐derived cellulose, where an environmentally‐unfriendly, harsh delignification procedure is necessary.^[^ ^63^ ^]^ Different loadings of *AHe*GH1 have been tested (1, 2, 5 and 10 mg g_matrix_^−1^) on two BC forms (i.e., powder and pellicles, **Figure** **1**); although 100% immobilization yield was observed till 5 mg g_matrix_^−1^ enzymatic loading for both the BC supports, the highest recovered activity (30%) was obtained employing 2 mg g_matrix_^−1^ of pure protein onto BC pellicles (see Supporting Information). The same enzymatic loadings onto BC powder resulted in a recovered enzymatic activity between 1%–10%, probably due to the nonuniform particle shape, porosity, and superficial area.^[^ ^62^ ^]^

Furthermore, while the powder presents challenging workability (e.g., difficult separation from the reaction medium as no filtration or centrifugation is possible), BC pellicles showed an easy isolation from the reaction environment, facilitating the downstream processing and demonstrating great potential for large‐scale biotransformations. After the selected immobilization was performed, the enzymatic leaking was analyzed by conventional techniques based on immobilized enzyme denaturation (e.g., 95 °C/10 min) followed by an SDS‐page analysis, demonstrating the covalent anchoring of the protein to the matrix.

**Figure 2:**
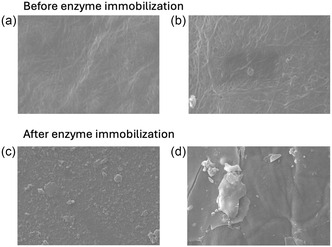
SEM images of BC pellicles before and after enzyme immobilization. a) untreated BC before immobilization (50× magnification); b) BC functionalized (APTES + glutaraldehyde); and c,d) BC after enzyme immobilization 20× and 50×, respectively.

To further characterize the carrier before and after enzyme immobilization, imaging analysis was carried out *via* scanning electron microscopy (SEM). Sheets supported with 2 mg g_matrix_^−1^ *AHe*GH1 did not show any surface changes with respect to the original non‐functionalized carrier (**Figure** **2**).

**Figure 3:**
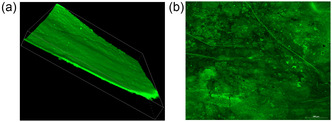
Confocal microscopy of BC pellicles immobilizing the fluorescein‐labeled *AHe*GH1 (2 mg g_matrix_^−1^): a) lateral snapshot and b) superficial snapshot (Magnification 20×).

Additionally, the spatial distribution of fluorophore‐labeled *AHe*GH1 was investigated. As observed in **Figure** **3** the biocatalyst was localized across the surface of cellulose, thus favoring a close contact with the substrate during the biotransformation.

The catalytic performance of the BC imm‐*AHe*GH1 has been assessed in batch reactions as previously described (i.e., substrate concentration 5 g L^−1^, biphasic media TMO:buffer 50:50, 40 °C). Complete conversion was observed in 7 h of reaction, corresponding to a three‐fold (3.23 g L^−1^ h^−1^) increase of STY, thus dramatically enhancing the productivity of PHL from PHZ (see Supporting Information). Noteworthy, the immobilized system afforded much higher activity than the free enzyme since the catalyst concentration (20 mg mL^−1^, enzyme loading: 2 mg g_matrix_^−1^) required for this reaction was 25 times lower than the free counterpart (1 mg mL^−1^). After catalyst removal and washing, the BC pellicle with imm‐*AHe*GH1 was used in several repeated batch reactions. More than 40% of molar conversion was observed after 13 cycles of biotransformation (7 h of reaction), thus demonstrating the robustness of the newly developed system. To compensate for the decreased activity, possibly due to a slow catalyst deactivation over time, a longer reaction (24 h) was assessed in the 14th cycle, restoring 95% of molar conversion (see Supporting Information).

To further increase the sustainability of the whole process, TMO has been recovered and, after appropriate analysis to monitor its purity (e.g., NMR technique), reused for different reaction cycles (>10). The sugar moieties were collected in the aqueous phase to be potentially reutilized for cell culture/cell feeding operations.^[^ ^65^ ^]^ As all the materials involved in the developed process can be recovered and reused, the designed strategy can be claimed as a zero‐waste reaction.

### 2.3 Bacterial Cellulose Production Using Apple Pomace as Fermentation Feedstock

To further enhance the valorization of apple pomace, we also studied the production of BC from this waste material, thus designing a bioprocess in which both the substrate and the immobilization support originated from the same feedstock. A medium rich in fermentable sugars (i.e., glucose ≈18 g L^−1^, sucrose ≈18 g L^−1^, fructose ≈54 g L^−1^) was obtained simply by homogenizing and centrifuging the apple pomace. Noteworthy, no further pretreatments of the sample were necessary to release nutrients. The static fermentation of the obtained medium with *K. xylinus* DSM 2325 led to the production of 4.03 ± 0.08 g L^−1^ of BC in 4 days without the addition of an external nitrogen source. This productivity was twice the one obtained using the conventional HS synthetic medium (2.00 ± 0.14 g L^−1^), which is commonly employed in BC production.

Regarding sugar consumption, glucose and sucrose were completely depleted by the end of fermentation, whereas fructose remained unused by the selected strain, with 54 g L^−1^ still present in the exhausted medium. Sugar concentrations were determined using the sucrose/D‐fructose/D‐glucose Assay Kit (K‐SUFRG, Megazyme Ltd.), as detailed in the Supplementary Information. Overall, these results highlight the possibility of using apple waste as fermentation feedstock for the easy obtainment of highly pure BC to be used in the immobilization trials of this study.

## 3 Conclusion

In this work, PHZ, one of the most abundant glycosyl flavonoids recoverable from apple‐processing waste, has been successfully biotransformed into the corresponding aglycone PHL, characterized by better antioxidant capacity, antibacterial properties, as well as ability to act as a skin penetration enhancer.

Keeping in mind the development of sustainable and cost‐effective processes, a BC immobilized halo‐thermophilic β‐glycosidase from *A. herbarius* (*AHe*GH1) has been employed together with biphasic systems (i.e., buffer:TMO 50:50, 20 mg mL^−1^ imm‐*AHe*GH1, 2 mg g_matrix_^−1^ enzyme loading, 5 g L^−1^ substrate), giving complete conversion after 7 h of reaction. While the enzyme immobilization onto BC pellicles allowed for high catalyst stabilization and its recovery and reuse, the employment of the unconventional green solvent TMO dramatically facilitated the isolation of the desired product (i.e., 95% of yield), leaving the sugar moieties in the water phase for their potential reutilization. BC not only exhibits unique characteristics as a protein immobilization carrier (e.g., high chemical purity, high crystallinity, and mechanical features), but also has been produced employing apple pomace as fermentation feedstock, further valorizing this residue.

Since all the materials involved in the biotransformations have been recovered and reused, the developed procedure can be considered appealing for its overall sustainability. Moreover, according to FDA and EMA regulations, the use of natural starting materials and biocatalytic approaches ensures the commercialization and labeling of the final product as natural as well, significantly impacting its market value.

## 4 Experimental Section

4.1

4.1.1

##### Chemicals and Reagents

Cell growing and strain maintaining media, as well as commercially available reagents, were purchased from Thermo Fischer Scientific or Merck (Sigma Aldrich). Organic solvents and chemical standards were bought from Merck (Sigma Aldrich). Merck Silica gel 60 F254 (aluminum foil) plates were used for TLC analysis; flash column chromatography was performed on Merck Silica gel (230–400 mesh). Detection of TLC analyses has been performed under UV light at 254 and 365 nm.

##### Cloning, Overexpression, and Purification of *AHe*GH1

Protein expression and purification were performed following previously reported protocols by Delgado et al.^[^ ^32^, ^33^ ^]^ (see Supporting Information).

##### Activity Assay of Free and imm‐*AHe*GH1

*AHe*GH1 free enzyme activity measurements were performed following previously reported protocols by Delgado et al.^[^ ^32^ ^]^ Specific activity was 23 U mg^−1^.

The residual activity measurement of the immobilized enzyme was performed as follows: 30 mg of substrate (*p*NPG, *p*‐nitrophenyl β‐D‐glucopyranoside) was dissolved in 10 mL of buffer HEPES 50 mM, pH 7.4. 100 mg of BC immobilized with the β‐glycosidase was added to start the reaction. The absorbance was read at 420 nm every 2 min. In the case of BC powder, every 2 min, a 2 mL sample was centrifuged for 30 s at 10 000 rpm. At this point, 1 mL of supernatant free of cellulosic material was transferred into a cuvette for the absorbance measurement. The imm‐*AHe*GH1 specific activity (U mg^−1^) is defined as μmol of *p*‐nitrophenol formed per minute per mg of immobilized enzyme. Specific activity was 6.9 U mg^−1^.

##### Bacterial Cellulose Production from HS Medium and Apple Waste

*Komagataeibacter xylinus* DSM2325 was maintained on GYC‐agar (10 g L^−1^ yeast extract, 50 g L^−1^ D‐glucose, 30 g L^−1^ calcium carbonate,15 g L^−1^ agar, pH 6.0) plates at 28 °C.

BC used as immobilization support was obtained by inoculating DSM2325 cells (2 OD_600 nm_) in Petri dishes (ID = 90 mm) using 20 mL of Hestrin–Schramm (HS) (20 g L^−1^ of glucose, 5 g L^−1^ of peptone, 5 g L^−1^ of yeast extract, 2.7 g L^−1^ of Na_2_HPO_4_, 1.15 g L^−1^ of citric acid, pH 6.0) or apple waste medium for 4 days under static condition at 28 °C. BC pellicles were collected and boiled in 0.5 M NaOH for 30 min to release entrapped cells in the matrix, washed with water, and rinsed with deionized water until neutral pH was reached. Finally, pellicles were dried at 40 °C/16 h.

##### Bacterial Cellulose Derivatization, *AHe*GH1 Immobilization

BC has been tested as a carrier for enzyme immobilization both in the form of pellicles and powder. To get BC powder, 100 mg of dried BC was first manually cut into smaller pieces and then homogenized in 100 mL of tetrahydrofuran (THF) under an ice bath (IKA T25 Digital ULTRA TURRAX‐ 3 cycles of 10 min, 15 000 rpm with 2 min break in between). Finally, the BC suspension was transferred into a round‐bottom flask, and the solvent was removed using a rotatory evaporator. Functionalization of BC pellicles and powder was performed according to de Souza et al.^[^ ^66^ ^]^ with small modifications. Briefly, in a 25 mL flask, 100 mg of BC was supplied with 10 mL of THF and 1 mL of APTES. The flask was left stirring for 4 h at room temperature; after centrifugation (4000 g for 10 min), THF was separated and 15 mL of phosphate buffer 50 mM, pH 7.0 was added. The suspension was well mixed and centrifuged, supernatant was discharged to get rid of any trace of residual THF. The procedure was performed twice. Finally, 10 mL of phosphate buffer 50 mM, pH 7.0 and 2 mL of glutaraldehyde 25% solution in water were added, and kept under stirring overnight. The functionalized BC was then washed three times with deionized water and stored in 50 mM phosphate buffer, pH 7.0, at 4 °C until used (see Supporting Information).

For *AHe*GH1 immobilization, in 50 mL tubes, 100 mg of support was added to 5 mL of potassium carbonate, 50 mM, pH 10.0. Then the enzyme solution was added to reach the tested loadings (1, 2, 5, 10 mg g_matrix_^−1^). The falcon was put on a rotatory bed until no residual protein was measured in the supernatant, assessed using Bradford assay (typically 16 h). Then, 1 mg mL^−1^ of sodium borohydride (NaBH_4_) was added and kept under stirring for 30 min. Finally, the immobilized enzyme was rinsed twice with distilled water, once with phosphate solution 50 mM, pH 5.0, and stored in HEPES 50 mM, pH 7.4 until used.

##### Sample Preparation for SEM and Confocal Analysis

After extensive washing with MilliQ water for salt removal, samples of BC pellicles immobilized with 2 mg g^−1^ of *AHe*GH1 were dried at 40 °C and subsequently lyophilized before the SEM analysis. Morphological investigation was carried out by field emission scanning electron microscopy using a SEM Leo 438 VP (Zeiss, Overcoached, Germany) operating at 20 kV and 3 × 10^−3^ Torr, secondary electron images (SEI) detection mode. Before analysis, samples were sputter‐coated with platinum to a thickness of ≈10 nm using a Leica EM ACE600 sputter coater (Leica Microsystems, Wetzlar, Germany) at 10 mA. For confocal analysis, a sample was fluorescently labeled according to the reported protocol by Velasco–Lozano et al.^[^ ^67^ ^]^ The enzyme has been immobilized onto BC functionalized pellicles as described above. Enzyme localization was observed using a Nikon A1 laser scanning confocal microscope with an excitation laser (λ: 488 nm) and the emission filter 500–550 nm for fluorescein signals.

##### Batch Reactions with Free or imm‐*AHe*GH1

Batch reactions using free or imm‐*AHe*GH1 were performed in 10/50 mL screw cap tubes; 2/10 mL reaction mixture composed of 50 mM HEPES buffer pH 7.4% and 10% DMSO, or 50 mM HEPES buffer pH 7.4/TMO 50:50, containing 5 g L^−1^ of phloridzin, 1 mg mL^−1^ of free enzyme/20 mg mL^−1^ of imm‐*AHe*GH1 (enzyme loading 2 mg g_matrix_^−1^), were left under gentle shaking at 40 °C. 100 μL aliquots were collected at different reaction times (30 min, 1, 2, 5, 24 h) for TLC analysis (EtOAc/Exane 9:1). After evaporation, the samples were resuspended in the mobile phase for HPLC analysis. The retention times were: phloridzin: 22.9 min, PHL: 29.8 min, confirmed by comparison with commercially available standards (see Supporting Information). Once the process was optimized, the organic phase derived from water extraction with EtOAc (buffer biotransformations) or TMO for biphasic systems was collected and evaporated. The crude extract was purified *via* flash chromatography (*n*‐hexane:EtOAc 7:3).

##### Apple Byproduct Treatment and Sugar Content Analysis

Apple waste medium was obtained following this procedure: wastes (cores, peels, and seeds) from 1 kg of apple “Golden Delicious” were blended with 125 mL of MilliQ water using a IKA T25 Digital ULTRA TURRAX in order to get a homogeneous suspension. After centrifugation to separate the solid debris at 15 000 rpm for 15 min, the supernatant was stored at −20 °C until used. Fresh samples were analyzed according to manufacturer instructions, using sucrose/D‐fructose/D‐glucose Assay Kit (K‐SUFRG), D‐xylose Assay Kit (K‐XYLOSE), and L‐arabinose/D‐galactose Assay Kit (K‐ARGA), all from Megazyme Ltd (see Supporting Information).

## Conflict of Interest

The authors declare no conflict of interest.

## Author Contributions

**Agostina Colacicco** and **Luca Nespoli** contributed equally to this work.

## Supporting information

Supplementary Material

## Data Availability

The data that support the findings of this study are available in the supplementary material of this article.
