# Supplementary material for: Valorization of Apple Pomace: Production of Phloretin Using a Bacterial Cellulose‐Immobilized β‐Glycosidase
Source: ChemSusChem. 2025 Apr 30;18(13):e202500592. doi: 10.1002/cssc.202500592 (PMC12231955; doi:10.1002/cssc.202500592)
Supplement: Supplementary file 1 — Supplementary Material [file CSSC-18-e202500592-s001.pdf]

Supplementary Information

Valorization of Apple Pomace: Production of Phloretin Using a Bacterial Cellulose-Immobilized β-Glycosidase

Agostina Colacicco, †[a] Luca Nespoli, †[a] Emma Ribul Moro,[a] Stefano Farris,[a] Francesco Molinari,[a] Diego Romano*[a] and Martina Letizia Contente*[a]

[a] Department of Food, Environmental and Nutritional Sciences (DeFENS), University of Milan, via Celoria, 2 Milan, Italy

e-mail: diego.romano@unimi.it
martina.contente@unimi.it

† These authors contributed equally to this work

Contents

1. *General information* ... 2
2. *Cloning, overexpression and purification of AHeGH1* ... 2
3. *Apple byproduct sugar content analysis* ... 3
4. *Immobilization efficiency of different enzyme loadings on the cellulose support* ... 3
5. *Chemical functionalization of bacterial cellulose via APTES and glutaraldehyde* ... 4
6. *HPLC analysis of biotransformations* ... 4
7. *Catalyst reuse* ... 5
8. *NMR spectra* ... 5
9. *References* ... 6

1. General information

NMR spectra were recorded on Brucker AvanceTM NEO 400 MHz spectrometer employing the residual signal of the deuterated solvent as internal standard. Chemical shifts (*δ*) are expressed in ppm and coupling constants (*J*) in Hertz (Hz). Organic solutions were concentrated using a Buchi rotary evaporator below 40 °C at 25 torr. Protein purification was carried out with ÄKTA Start purifier (GE Healthcare) and 1 mL His-Trap IMAC (GE Healthcare) columns. HPLC analyses were performed using a Merck-Hitachi LaChrom Liquid Cromatograph with L-7200 autosampler, L-7100 pump and L-7400 UV- detector. Analyses were carried out using the following gradient: 90/10 (v/v) H_2O milliQ/ACN until 60% of ACN for 30 min (t0min → t30min); reaching 100% ACN at 35 min (t30min → t35min), maintaining 100% ACN for 5 min (t35min → t40min); λ = 280 nm; flow rate: 0.7 mL/min; column: LiChroCART (250 x 4.6 mm x 5 µm).

2. Cloning, overexpression and purification of *AHe*GH1

Protein expression and purification were performed following previously reported protocols by Delgado *et al.*[1-2] Figure S1 shows pellet, crude extract, flow through fractions and pure protein analyzed by SDS-PAGE. The monomer of HE is 52.1 kDa. Typically, starting from 1 L culture it is possible to obtain 55 mg of pure protein.

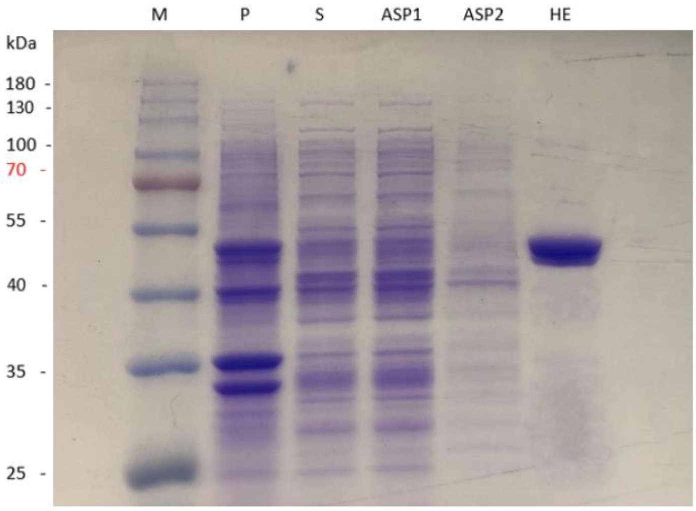

Figure S1: M: marker, P: pellet, S: crude extract, ASP1, ASP2: flow through fractions, HE: *AHe*GH1 pure protein.

3. Apple byproduct sugar content analysis

After appropriate treatment, fresh samples were analyzed according to manufacturer instructions, using sucrose/D-fructose/D-glucose Assay Kit (K-SUFRG), D-xylose Assay Kit (K-XYLOSE), and L-arabinose/D-galactose Assay Kit (K-ARGA), all from Megazyme Ltd.

Sugar	Concentration
D-glucose	18.27 g/L
Sucrose	18.54 g/L
D-fructose	53.66 g/L
D-xylose	0.85 g/L
L-arabinose	Not detectable
D-galactose	Not detectable

4. Immobilization efficiency of different enzyme loadings on the cellulose support

Different enzyme loadings (1 mg/g_{matrix}, 2 mg/g_{matrix}, 5 mg/g_{matrix} , and 10 mg/g_{matrix}) were analyzed simultaneously to identify the best performing immobilization system on cellulose pellicles. For the 10 mg/g_{matrix} loading a immobilization yield of 52% has been detected, Therefore, the 10 mg/g_{matrix} loading was discarded from subsequent activity analysis.

Type of immobilization support	Enzyme loading	Enzymatic activity
Cellulose pellicles	1 mg/g_{matrix}	2.6 U/mg
	2 mg/g_{matrix}	6.9 U/mg
	5 mg/g_{matrix}	2.11 U/mg

The same experiments were carried out on BC powder obtaining a recovered enzymatic activity between 0.2 -1.7 U/mg.

5. Chemical functionalization of bacterial cellulose via APTES and glutaraldehyde

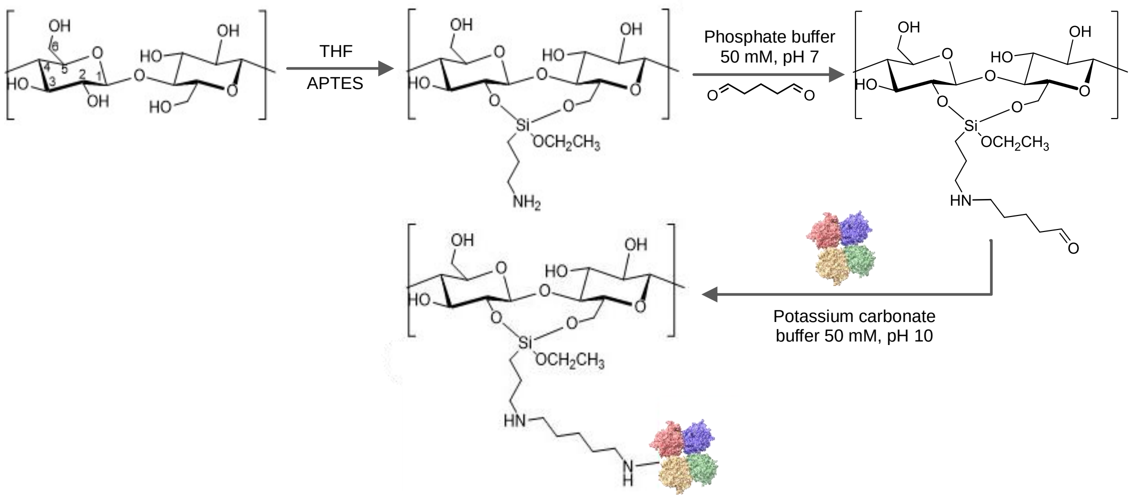

Figure S2: Process of chemical functionalization of bacterial cellulose via APTES (3-aminopropyltriethoxysilane) and glutaraldehyde, and subsequent immobilization of A*He*GH1 on the support.

6. HPLC analysis of biotransformations

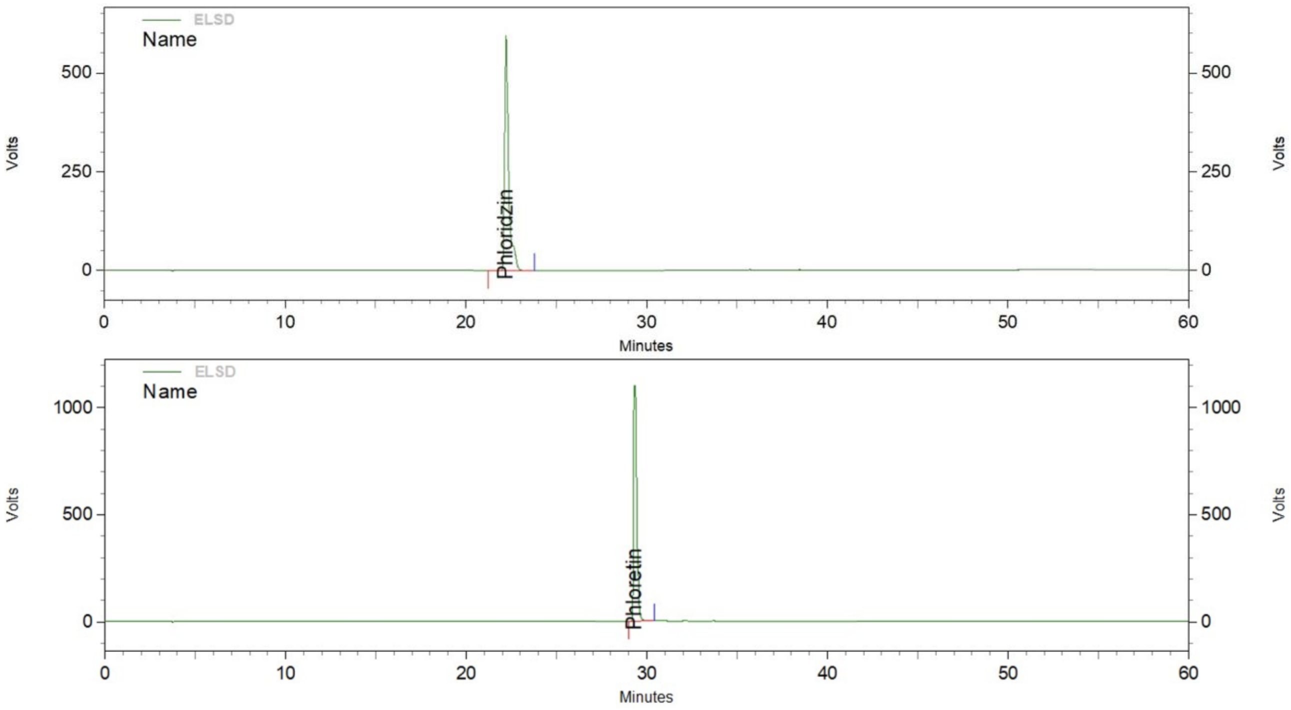

Figure S3. HPLC chromatograms of commercial standards: phloridzin (above) and phloretin (below).

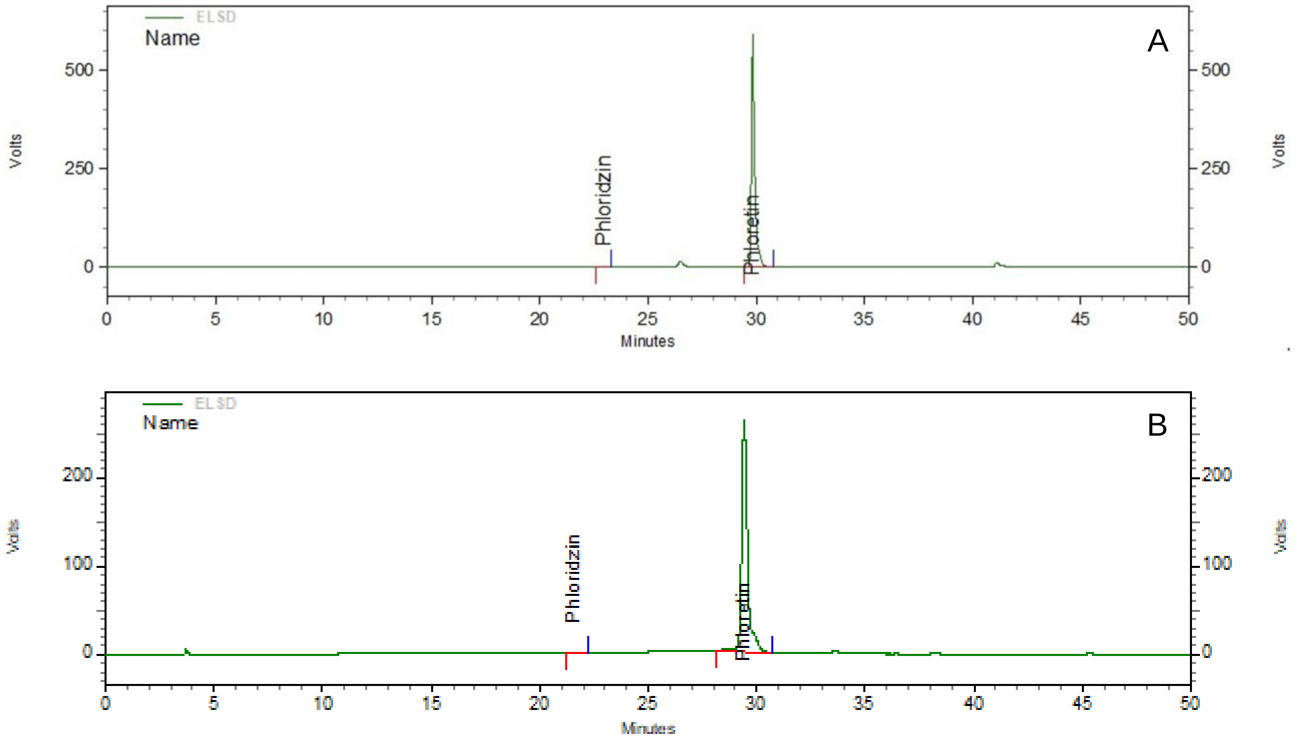

Figure S4. HPLC chromatograms of the biotransformation performed by the immobilized enzyme *AHe*GH1 at 24 hours (water medium biotransformation) (A) and at 7 hours biphasic (water/TMO 50:50) (B).

7. Catalyst reuse

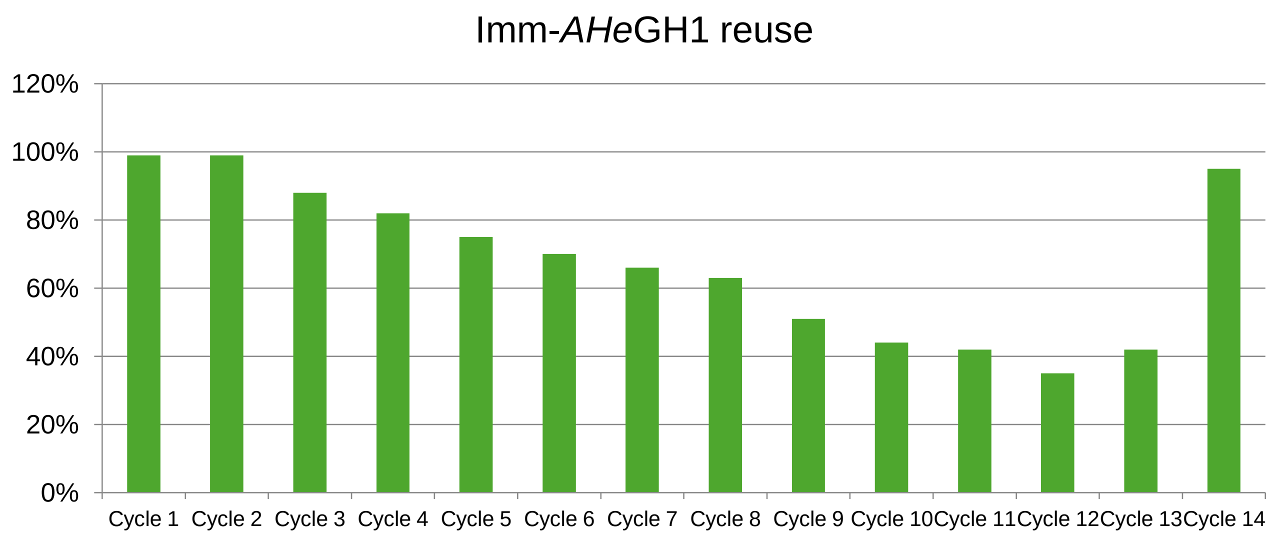

Figure S5. Imm-*AHe*GH1 reuse. Y axis: PHL molar conversion after 7 h of reaction; X axis: number of cycles**.** In cycle 14 the conversion was monitored after 24 h or reaction, restoring the initial full conversion.

8. NMR spectra

Phloretin: ^{1}H NMR (400 MHz, DMSO-*d*6) δ (ppm): 7.03 (d, *J* = 8.6 Hz 1H), 6.67 (d, *J* = 8.6 Hz 1H), 5.82 (s, 2H), 3.23 (t, *J* = 8.0 1H), 2.78 (t, *J* = 7.7 Hz, 1H); ^{13}C-NMR (150 MHz, DMSO- d_6) δ (ppm): 204.7, 165.1, 164.7, 155.9, 132.1, 129.6, 115.5, 104.2, 95.1, 45.9, 29.9.

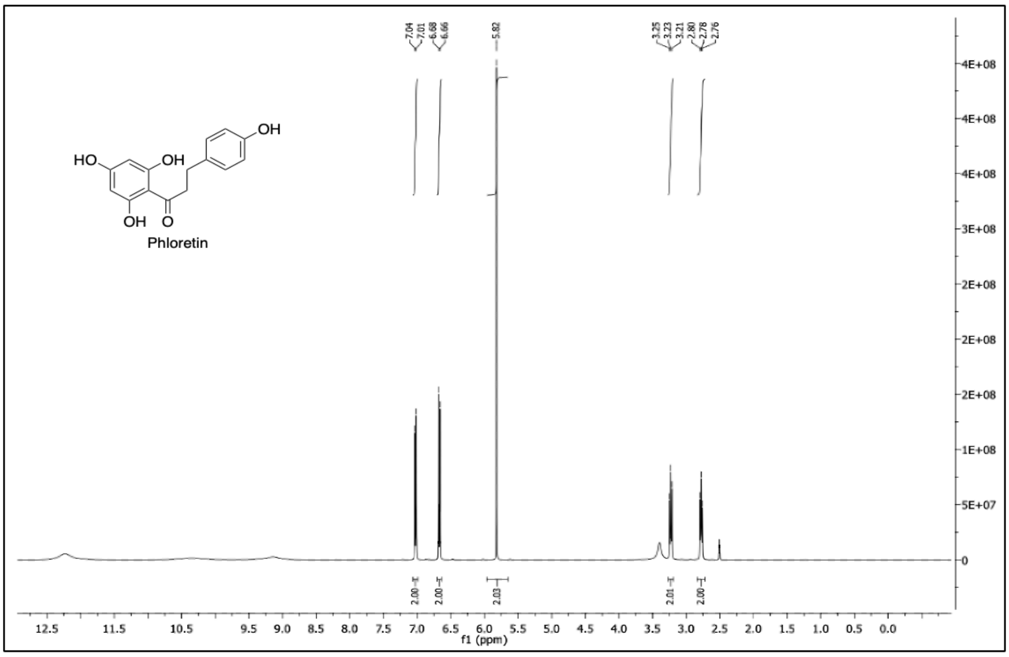

Figure S6: ^{1}H-NMR Phloretin

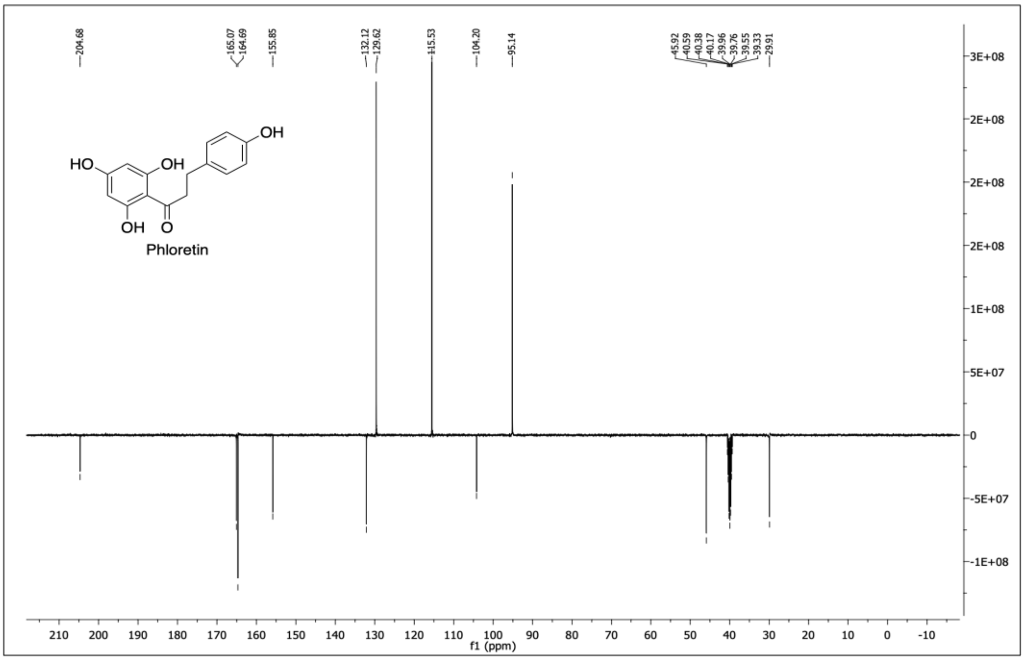

Figure S7: ^{13}C-NMR Phloretin

9. References

1. L. Delgado, M. Parker, I. Fisk and F. Paradisi, *Food Chem.* **2020**, *323*, 126825.
2. L. Delgado, C. M. Heckmann, F. Di Pisa, L. Gourlay, F.Paradisi *Chembiochem* **2021**, *22*, 1223.
